# Molecular Insight into the Processes and Mechanisms of N_2_ Adsorption and Accumulation at the Hydrophobic Solid/Liquid Interface

**DOI:** 10.3390/molecules29112711

**Published:** 2024-06-06

**Authors:** Bao Li, Dan Su

**Affiliations:** College of Mining Engineering, Taiyuan University of Technology, Taiyuan 030024, China

**Keywords:** nano-gas layers, molecular dynamics simulations, N_2_ molecules, solid–liquid interface, adsorption

## Abstract

In this study, molecular dynamics (MD) simulations were employed to elucidate the processes and underlying mechanisms that govern the adsorption and accumulation of gas (represented by N_2_) at the hydrophobic solid–liquid interface, using the GROMACS program with an AMBER force field. Our findings indicate that, regardless of surface roughness, the presence of water molecules is a prerequisite for the adsorption and aggregation of N_2_ molecules on solid surfaces. N_2_ molecules dissolved in water can cluster even without a solid substrate. In the gas–solid–liquid system, the exclusion of water molecules at the hydrophobic solid–liquid interface and the adsorption of N_2_ molecules do not occur simultaneously. A loosely arranged layer of water molecules is initially formed on the hydrophobic solid surface. The two-stage process of N_2_ molecule adsorption and accumulation at the hydrophobic solid/liquid interface involves initial adsorption to the solid surface, displacing water molecules, followed by N_2_ accumulation via self-interaction after saturating the substrate’s surface. The process and underlying mechanisms of gas adsorption and accumulation at hydrophobic solid/liquid interfaces elucidated in this study offer a molecular-level understanding of nano-gas layer formation.

## 1. Introduction

Nano-gas layers, which are quasi-two-dimensional gaseous aggregates at solid–liquid interfaces, have attracted extensive attention for more than ten years. Utilizing experimental techniques such as the tapping-mode atomic force microscope (TM-AFM), the stability, structural ordering, and preparation methods of nano-gas layers have been extensively studied, confirming that they hold significant applications in interface science [1,2,3,4,5]. Various studies have indicated that their presence could enhance interfacial slip lengths [1], induce protein folding [2], facilitate the nucleation of gas hydrates [3], and impact electrochemical reactions at interfaces [4,5].

In 2007, Hu et al. utilized the tapping mode of atomic force microscopy to observe gaseous layers on the surface of highly oriented pyrolytic graphite (HOPG), and these layers were not easy to form or gradually disappeared in degassed water [6]. Following the initial discovery, research on nano-gas layers progressively emerged, primarily focusing on the fundamental properties and behaviors of these layers and providing theoretical explanations. Nguyen et al. demonstrated through AFM force curves that nanobubbles on HOPG surfaces do not exist in isolation but are typically surrounded by gas layers [7]. Schlesinger et al. [8] investigated the state of gas layers near hydrophobic surfaces in water with varying degrees of gas saturation, mapping out three-dimensional structures of gaseous substances near gas–liquid interfaces. The results indicated that hydrophobic surfaces commonly exhibit gas enrichment layers with heights of 2–5 nm. Notably, these layers showed a weak dependence on the solubility of gases in water. These results confirm the widespread presence of these layers on hydrophobic interfaces, characterized by a non-uniform distribution of gas density that decreases with increasing distance. Additionally, the thickness of these layers is influenced by the gas content in the liquid.

The alcohol water replacement method involves using low-gas-solubility pure water to replace high-gas-solubility alcohols (typically ethanol) near the gas–liquid interface, creating a transient state of gas supersaturation and consequently generating nano-gas layers [9]. While this alcohol water replacement method allows for the visualization of nano-gas layers on hydrophobic surfaces, it is not an efficient method for their production. Understanding the conditions for the formation of nano-gas layers is a prerequisite for developing efficient preparation methods. Zhang et al. [10] investigated the formation of nano-gas layers on various substrates such as MoS_2_, talc, HOPG, and silicon, revealing that the conditions for nano-gas layer formation are stringent and influenced by the hydrophobicity of the surface, the smoothness of the material, and the presence of an ordered crystalline structure. Nevertheless, the scarcity of experimental techniques has concurrently limited further understanding of the formation of gas layers.

Molecular dynamics (MD) simulations, characterized by their femtosecond (fs) time resolution and the ability to resolve the motion of all atoms, are extensively utilized in studying dynamic and structural behaviors in various processes. Weijs et al. [11], through MD simulations, observed that nanobubbles spontaneously nucleate in heavily gas-supersaturated liquids and migrate toward the surface. Sendner et al. [12] simulated several specific gases (Ar, Ne, O_2_, and an “ideal” gas type “X”) dissolved in a water model on a diamond-like structure. Their findings highlighted gas enrichment within approximately 1 nm of a hydrophobic surface for all investigated gas types. The above studies demonstrate that the inherent spatial resolution of MD simulations is sufficiently high to elucidate the mechanisms of gas adsorption and accumulation.

In a previous study [13], we focused on investigating the impact of grooved surfaces with varying heights and widths on the adsorption and accumulation of N_2_ at the hydrophobic solid–liquid interface through MD simulations. The results demonstrated that an increase in the surface roughness of the solid substrate facilitated the adsorption and accumulation of N_2_. However, to date, there has been a lack of comprehensive research in the processes that govern the adsorption and accumulation of gas at the hydrophobic solid/liquid interface. Understanding the behavior of gas molecules at interfaces can lay the groundwork for the further exploration of the properties of nano-gas layers and guide the development of preparation methods, thereby driving their applications across various fields such as materials science, surface wetting, and biomedicine.

In this study, two gas–solid models (smooth solid and rough solid surfaces) and a gas–liquid model were developed to evaluate the significance of water in gas adsorption and accumulation. Subsequently, a gas–liquid–solid three-phase model was constructed to provide a comprehensive explanation of the gas adsorption and accumulation at the solid–liquid interface, including aspects such as the adsorption configuration, the formation of a loosely arranged water molecular layer, the density distribution, and the interaction energy. This study can provide fundamental insights into the gas adsorption and accumulation processes involved in the formation of nano-gas layers, as well as the changes in the interactions among the gas–liquid–solid phases during these processes.

## 2. Results and Discussion

### 2.1. Gas–Solid and Gas–Liquid Systems

**Figure 1:**
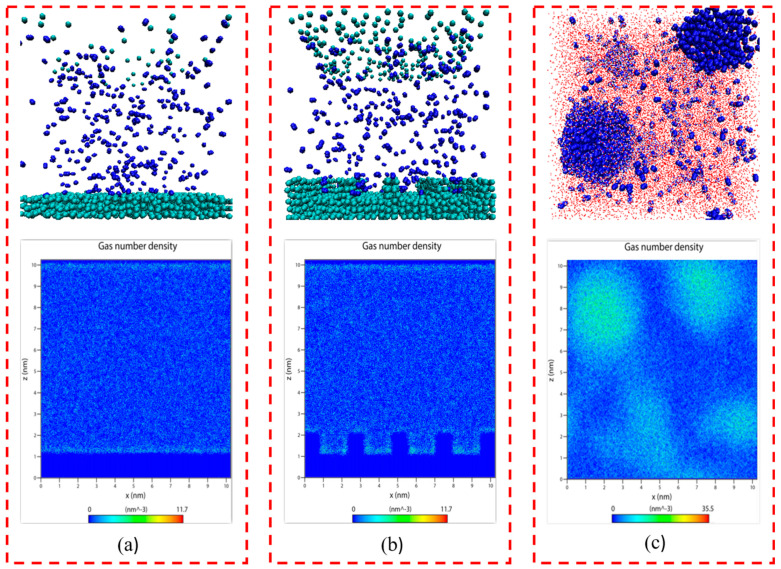
Snapshots of the equilibrium model: (**a**) gas–solid model (smooth substrate); (**b**) gas–solid model (rough substrate); (**c**) gas–liquid model. The colors of the atoms are as follows: C, green; H, red; O, white; N, blue.

It is evident from Figure 1a,b that when the system reached equilibrium, there was only little adsorption or aggregation of N_2_ molecules on either smooth or rough substrates in the gas–solid model, with most of the N_2_ molecule remaining uniformly distributed in the simulated box. This indicates that in the absence of water, it is difficult for N_2_ molecules to adsorb or aggregate on solid substrates, regardless of their roughness, signifying that the presence of water molecules is a prerequisite for the extensive adsorption and aggregation of N_2_ molecules on solid surfaces.

As shown in Figure 1c, N_2_ molecules that were initially uniformly dispersed in water showed aggregation at various locations. This indicates that N_2_ molecules that were dissolved in water can cluster even without a solid substrate, consistent with the formation of bulk phase micro-nanobubbles in solutions under ultrasound or intense shearing conditions.

### 2.2. Gas–Solid–Liquid System

#### 2.2.1. N_2_ Molecular Adsorption Process and Adsorption Configuration

**Figure 2:**
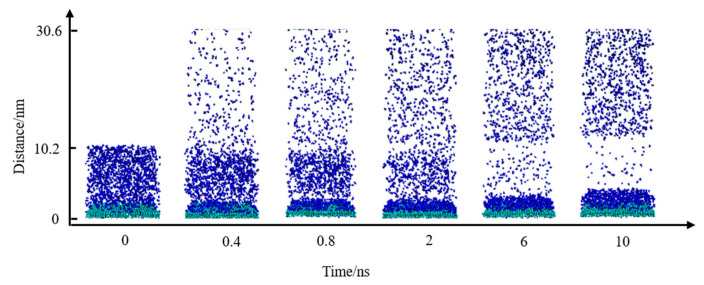
Snapshots of N_2_ molecules’ adsorption process. The colors of the atoms are as follows: C, green; N, blue.

In the initial model, N_2_ molecules were randomly placed within the simulation cell. To demonstrate the adsorption and accumulation of N_2_ molecules, H_2_O molecules were hidden in the system, and snapshots at various simulation times were extracted, as shown in Figure 2. It is observed that from 0 to 6 ns, the N_2_ molecules in water increasingly migrated and adhered to the substrate surface, with the remaining N_2_ molecules dispersing into the vacuum layer above the aqueous phase.

**Figure 3:**
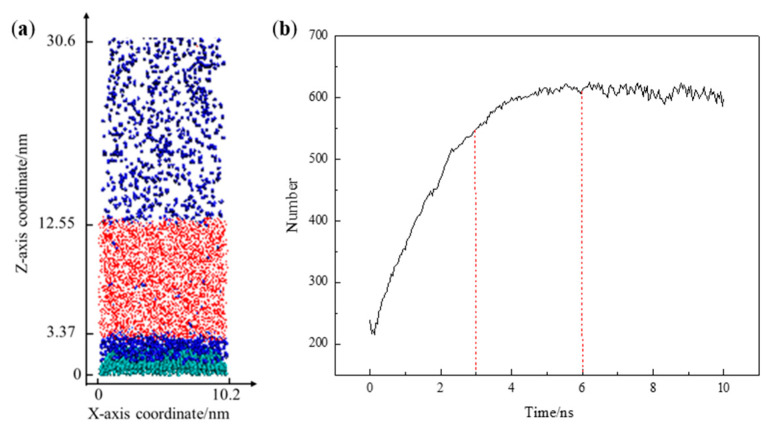
(**a**) Snapshot of the equilibrium configuration; (**b**) the number of N_2_ molecules as a function of the simulation time. The colors of the atoms are as follows: C, green; H, red; O, white; N, blue.

Figure 3a illustrates the adsorption equilibrium configuration of the solid–liquid–gas three-phase simulation system. Following the establishment of adsorption equilibrium, N_2_ molecules were adsorbed and accumulated within the range of 0 < Z < 3.37 nm at the solid–liquid interface. Figure 3b shows the quantity of N_2_ molecules within this range as a function of the simulation time. There is a minimum in the number of N_2_ molecules within the range of 0–0.5 ns. This phenomenon results from the specific number of N_2_ molecules (approximately 400–450) accommodated at the solid–liquid interface during the model construction process, along with their uniform distribution. At the beginning of the simulation, the hydrogen bond interactions among H_2_O molecules are more significant than interactions between N_2_ and H_2_O molecules, resulting in the separation of N_2_ and H_2_O molecules [13]. As the simulation time increases, there is a rapid rise in the number of N_2_ molecules adsorbed at the solid–liquid interface.

During the simulation period of 3–6 ns, the growth rate of the curve representing the quantity of adsorbed N_2_ molecules experiences a slowdown. This suggests a gradual decrease in the rate of increase in the number of N_2_ molecules adsorbed at the solid–liquid interface, likely due to the adsorption sites on the substrate surface becoming occupied with increasing simulation time, with the accumulation process dominated by N_2_ molecule interactions becoming slower. When the simulation time exceeds 6 ns, the number of N_2_ molecules at the solid–liquid interface no longer changes significantly, indicating that a dynamic equilibrium between adsorption and desorption has been reached. At this juncture, the N_2_ molecules that were initially uniformly distributed in water were found in three locations: some aggregated at the solid–liquid interface, a minimal number were still present in the aqueous phase, and there was an excess in the vacuum layer of the simulation. It is worth noting that the minimal N_2_ molecules remaining in the water phase could be due to hydrogen bond (O–H···N) formations between H_2_O and N_2_ molecules [14]. N_2_ molecules in the vacuum are attributed to the over-saturation of nitrogen molecules during model construction. As the simulation progresses, the escape of excess N_2_ molecules from the aqueous phase under atmospheric pressure conditions occurs.

#### 2.2.2. Formation of a Loosely Arranged Water Molecular Layer at the Solid–Liquid Interface

**Figure 4:**
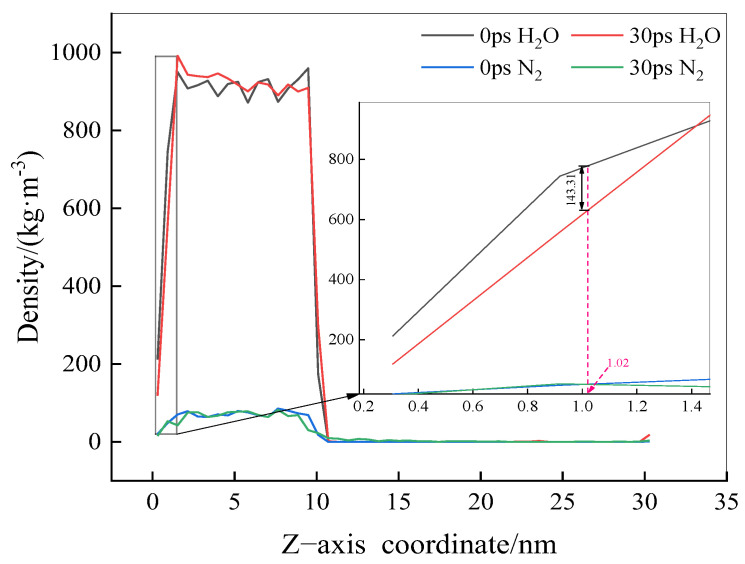
Density distribution of H_2_O and N_2_ molecules at 0 ps and 30 ps.

To investigate how N_2_ molecules displace pre-adsorbed water molecules at the solid–liquid interface, resulting in adsorption and accumulation, the density curves of H_2_O and N_2_ molecules along the Z-axis were extracted at 0 and 30 ps, as shown in Figure 4. It is observed that at a simulation time of 30 ps, the density of water molecules at the solid–liquid interface (at Z = 1.02 nm) is markedly lower than in the initial model (at 0 ps), with a difference of approximately 143.31 kg/m^3^. In contrast, the density of N_2_ molecules shows no significant change between 0 and 30 ps. This indicates that the exclusion of water molecules at the solid–liquid interface and the adsorption of N_2_ molecules do not occur simultaneously.

Due to the hydrophobic interaction of the solid surface, the density of H_2_O molecules at the interface decreases, forming a loosely arranged layer of water molecules. As the simulation time increases, N_2_ molecules gradually adsorb onto the solid surface, continuously displacing water molecules and eventually accumulating at the solid–liquid interface, forming a gaseous adsorption layer. Previous studies have shown that when hydrophobic solids are immersed in water, the density of water molecules at their surface is lower than that of bulk water molecules, extending from a few Å to several nm [15,16], and the characteristics of this low-density water layer are closely related to the hydrophobicity of the solid surface. These observations agree with the findings of the current study.

#### 2.2.3. Density Distribution

**Figure 5:**
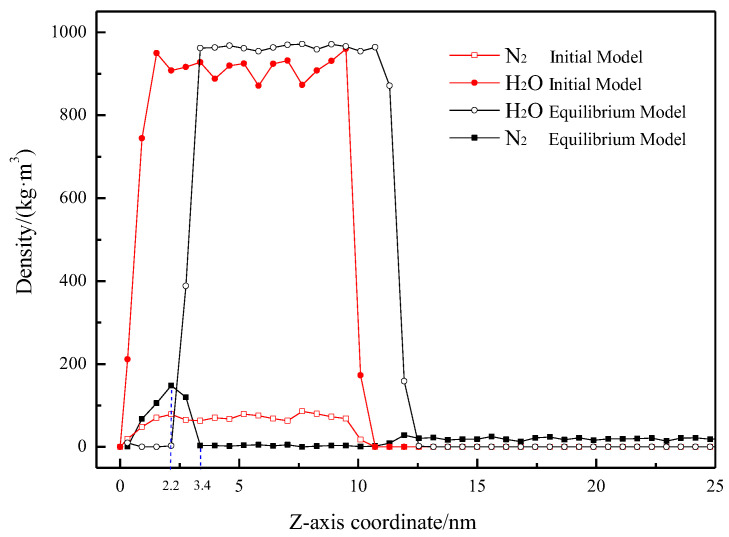
Density profiles for H_2_O and N_2_ in initial and equilibrium models as a function of the distance Z.

To clearly depict the distribution of H_2_O and N_2_ in the system before and after the simulation, density distribution curves of N_2_ and H_2_O were plotted, as shown in Figure 5, with the origin at the solid surface and the variable being the height perpendicular to this surface. It is obvious that the average density of the water phase before and after simulation equilibrium is close to 950 kg/m^3^, which is close to the actual density of water (970 kg/m^3^), indicating that the model construction, force field selection, and parameter settings of the simulation system are reasonable.

In the initial model, due to the uniform distribution of N_2_ in water, the distribution of H_2_O and N_2_ perpendicular to the solid surface is consistent (distribution range is approximately 0–10.5 nm). The density of N_2_ in the initial stage is about 73.07 ± 6.21 kg/m^3^. Correspondingly, after simulation equilibrium, the density of H_2_O molecules is zero within the 0–2.2 nm range, and the maximum density of N_2_ molecules is 147.97 kg/m^3^. Following adsorption equilibrium, the distribution range of N_2_ molecules at the solid–liquid interface is approximately 0–3.4 nm, consistent with the height of the gas-rich layer depicted in Figure 2 and Figure 3. This indicates that the H_2_O molecules at the solid–liquid interface have been displaced, and N_2_ molecules have undergone adsorption and accumulation. Additionally, the density of the N_2_ molecular layer at the hydrophobic solid–liquid interface is significantly higher than the density of N_2_ molecules dissolved in water, aligning with the high-density feature of nanobubbles [17].

#### 2.2.4. Interaction Energies

**Figure 6:**
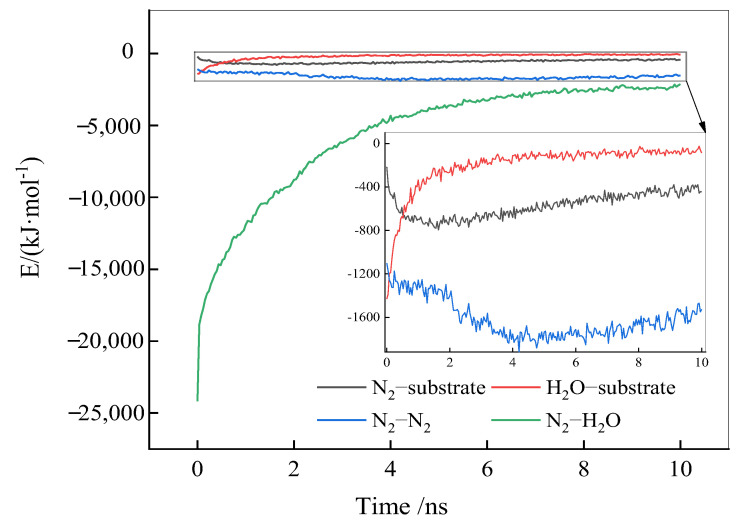
Interaction energies as a function of the simulation time.

The adsorption and accumulation of N_2_ molecules at the solid–liquid interface involve interactions between N_2_, H_2_O, and the substrate surface; N_2_-N_2_; and N_2_-H_2_O. Figure 6 illustrates these interaction energies as a function of the simulation time. A negative interaction energy indicates a stable interaction between object phases, and the more negative the interaction energy, the more favorable the interaction.

From Figure 6, as the simulation progresses, the interaction energy between N_2_ and H_2_O molecules decreases sharply, indicating a gradual separation of N_2_ and H_2_O molecules from a uniform mixed distribution to individual aggregation. At the same time, the interaction energy between N_2_ molecules and the substrate surface significantly increases, the interaction energy between H_2_O molecules and the substrate surface decreases, and the interaction energy among N_2_ molecules remains largely unchanged, suggesting that in this stage, N_2_ molecules primarily adsorb to the solid surface through interactions with the substrate, without significant clustering among N_2_ molecules. As the simulation progresses, the substrate surface becomes fully occupied by N_2_ molecules, increasing the interaction energy among N_2_ molecules and leading to their aggregation. In the later stages of the simulation, the reduction in interaction energy between N_2_ and the substrate surface, and among N_2_ molecules themselves, is likely due to an excess of N_2_ molecules escaping from the solid–liquid interface into the vacuum layer.

To summarize, the adsorption and accumulation of N_2_ molecules at the hydrophobic solid–liquid interface could be bifurcated into two stages. In the first stage, N_2_ molecules adsorb to the solid surface through their interaction with the substrate, continually displacing water molecules at the solid–liquid interface. Once all the adsorption sites on the substrate surface are occupied, the second stage involves the accumulation of N_2_ molecules through interactions among themselves.

## 3. Models and Simulations

### 3.1. Models

**Figure 7:**
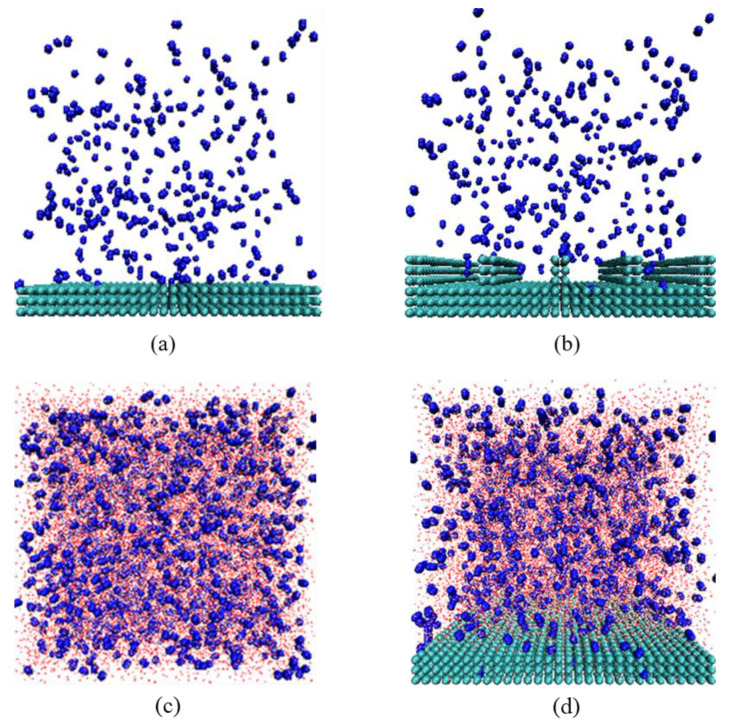
Snapshot of gas–solid model: (**a**) smooth substrate, (**b**) rough substrate, (**c**) gas–liquid model, (**d**) gas–solid–liquid model. The colors of the atoms are as follows: C, green; H, red; O, white; N, blue.

In this study, N_2_ molecules were selected as a representative gas, and H_2_O molecules were modeled using a three-point charge SPC water model. Carbon atoms were selected to construct the solid model. Approximately 3000 carbon atoms were arranged in three layers within an area of 10.20 × 10.20 nm^2^, with the equilibrium distance between carbon atoms set to 0.34 nm, representing the hydrophobic solid surface. To assess the conditions for gas adsorption and accumulation, two gas–solid models and a gas–liquid model were constructed. For the former, a smooth substrate representation was achieved using three layers of carbon atoms. To simulate the variation between smooth and rough substrates, grooves were added above these carbon atom layers, effectively defenestrating the groove substrate surface characteristics. These substrates were placed at the bottom of a 10.20 × 10.20 × 10.20 nm^3^ simulation box, with 300 N_2_ molecules added, forming the initial models as shown in Figure 7a,b. For the gas–liquid model, 2000 N_2_ molecules and 35,020 H_2_O molecules were randomly distributed in a 10.20 × 10.20 × 10.20 nm^3^ simulation box, with a water molecule density of 33 molecules per cubic nanometer, as depicted in Figure 7c.

To investigate the adsorption and accumulation processes of N_2_ molecules at the solid–liquid interface, the aforementioned carbon layers were positioned inside a 10.20 × 10.20 × 10.20 nm^3^ simulation box. A total of 1500 N_2_ molecules were added, and the remaining space was filled with SPC water molecules, totaling 29,715 H_2_O molecules, as shown in Figure 7d. To eliminate the impact of periodicity, a 20.40 nm vacuum layer was implemented at the top of the simulation box [18,19,20].

### 3.2. Simulation Details

MD simulations were conducted using GROMACS2018.8 software, employing an AMBER force field to characterize the bonding and non-bonding interactions between the analyzed molecules [21,22]. The computation of interatomic non-bonding interactions was based on the Lennard–Jones potential functions [23]. The particle mesh Ewald (PME) method was applied to compute long-range electrostatic interactions with a cut-off of 1.0 nm [24]. The system initialization involved energy minimization down to 1000 kJ mol^−1^ nm^−1^ using the steepest descent method. After energy minimization, MD simulations proceeded in the NVT ensemble with a 2.0 fs time step, and temperature control at 298 K was maintained using a Nosé–Hoover thermostat [25,26,27]. The Parrinello–Rahman pressure coupling method was implemented to ensure that the pressure of the entire system was maintained at 1.01 × 10^5^ Pa. Periodic boundary conditions were set in all directions across all simulation systems. A trajectory file was obtained every 4 × 10^4^ fs for subsequent kinetic analysis, and VMD1.9.3 software was utilized to visualize the system’s microstructure [28]. 

## 4. Conclusions

The study provides in-depth insights into the processes of N_2_ adsorption and accumulation at the hydrophobic solid–liquid interface. The main conclusions obtained are as follows:

(1) Regardless of the surface’s roughness, the presence of water molecules is a prerequisite for the adsorption and aggregation of N_2_ molecules on solid surfaces. N_2_ molecules dissolved in water can cluster even without a solid substrate. 

(2) In the gas–solid–liquid system, the exclusion of water molecules at the solid–liquid interface and the adsorption of N_2_ molecules do not occur simultaneously. A loosely arranged layer of water molecules is initially formed on the hydrophobic solid surface. 

(3) The two-stage process of N_2_ molecule adsorption and accumulation at the hydrophobic solid-liquid interface involves initial adsorption to the solid surface, displacing water molecules, followed by N_2_ accumulation via self-interaction after saturating the substrate’s adsorption sites.

It is important to note that this study utilizes MD simulations to elucidate the process and underlying mechanisms of gas adsorption and accumulation at hydrophobic solid/liquid interfaces at the molecular level. However, the models constructed in this study are only based on hydrophobic surface models and only employ nitrogen as a representative gas. This study lacks an investigation into the hydrophilic model, types, and concentrations of gases. Further exploration is warranted in subsequent studies.

## Data Availability

Data are contained within the article.
